# The 2D:4D-Ratio and Neuroticism Revisited: Empirical Evidence from Germany and China

**DOI:** 10.3389/fpsyg.2016.00811

**Published:** 2016-06-10

**Authors:** Cornelia Sindermann, Mei Li, Rayna Sariyska, Bernd Lachmann, Éilish Duke, Andrew Cooper, Lidia Warneck, Christian Montag

**Affiliations:** ^1^Institute of Psychology and Education, Ulm UniversityUlm, Germany; ^2^Student Counseling Center, Beijing University of Civil Engineering and ArchitectureBeijing, China; ^3^Department of Psychology, Goldsmiths, University of LondonLondon, UK; ^4^Key Laboratory for NeuroInformation/Center for Information in Medicine, School of Life Science and Technology, University of Electronic Science and Technology of ChinaChengdu, China

**Keywords:** 2D:4D, Finger Ratio, Personality, Big Five, Neuroticism, Cross Cultural, China, Germany

## Abstract

The 2D:4D-Ratio, as an indirect measure of the fetal testosterone to estradiol ratio, is potentially very important for understanding and explaining different personality traits. It was the aim of the present study to replicate the findings from Fink et al. (2004) about the relation between individual differences in 2D:4D-Ratios and the Five Factor Model in different cultural groups. Therefore a sample of *n* = 78 Chinese and *n* = 370 German participants was recruited. Every participant provided hand scans of both hands, from which 2D:4D-Ratios were computed. Moreover, all participants filled in the NEO Five Factor Inventory (NEO-FFI). Significant sex differences were found for ratios of both hands in the expected direction, with females showing higher ratios than males. With respect to links between personality and the digit ratio, a positive association was observed between 2D:4D-Ratio and Neuroticism in females, as shown in the earlier study. These findings were observed in both female subsamples from China and Germany, as well as in the full sample of participants. But in contrast to the results for the whole and the German female sample, where 2D:4D-Ratio of both hands were related to Neuroticism, in the Chinese female sample only left hand 2D:4D-Ratio was significantly and positively related to Neuroticism. There were no significant correlations found in any of the male samples. Thus, prenatal exposure to sex steroids appears to influence the personality factor Neuroticism in females specifically. This finding potentially has implications for mental health, as Neuroticism has been shown to be a risk factor for various forms of psychopathology.

## Introduction

Personality traits are often considered stereotypically male, for example aggressive behavior or sensation seeking, and stereotypically female, for example anxiety and Neuroticism (Buss and Perry, 1992; Feingold, 1994; Weisberg et al., 2011). But not all men are aggressive and not all women are anxious; individuals across both sexes simply vary on these dimensional traits, and will display both characteristically male and female behaviors, given a specific situation and their trait level. In other words, there is substantial variability in personality traits both between and within sexes. The aim of the present study was to revisit an often investigated biomarker in traits linked to sexual dimorphisms, namely the 2D:4D-Ratio, which has been widely identified as an indirect marker of prenatal testosterone levels.

The 2D:4D-Ratio describes the relative length of the index (2^nd^) finger to the ring (4^th^) finger. It is a sex dimorphic trait, with males tending to have smaller ratios than females. As mentioned above, it is often used as an indirect measure of prenatal testosterone concentration (Hönekopp and Watson, 2011; Manning and Fink, 2011). The smaller the index (2^nd^) finger compared to the ring (4^th^) finger – resulting in a smaller ratio – the higher the relative proportion of prenatal testosterone relative to prenatal estradiol. This pattern indicates a more “male” hand. A higher ratio, due to a longer index (2^nd^) finger relative to the ring (4^th^) finger, indicates a more “female” hand, and is associated with a lower prenatal testosterone to estradiol ratio (Lutchmaya et al., 2004; Manning et al., 2014).

Existing literature suggests that this is due to the action of groups of Homeobox (Hox) genes. These genes appear to regulate the ontogeny of the reproductive system, as well as the growth and patterning of fingers (Kondo et al., 1997). Based on previous findings, Manning et al. (1998) examined the association between the 2D:4D-Ratio and exposure to prenatal sex steroids. They suggested that Hox genes control development of the digits and male testes. Furthermore, they pointed out that the testosterone developed in the testes of the male fetus affects the development of digits, including a lower 2D:4D-Ratio, via higher concentrations of fetal testosterone (Manning et al., 1998). They also found associations between the 2D:4D-Ratio and prenatal testosterone in females. They suggested that these relationships were based on the influence of maternal testosterone on the development of the fingers of the unborn child (Manning et al., 1998). The negative association between concentrations of prenatal testosterone and 2D:4D-Ratio has been widely replicated in males and females (for examples see Manning et al., 1998; Lutchmaya et al., 2004). And moreover an association between 2D:4D-Ratio and prenatal exposure to oestrogenic and antiandrogenic compounds and between 2D:4D-Ratio and prenatal androgen and estrogen signaling could be found in two experimental studies on mice and rats (Zheng and Cohn, 2011; Auger et al., 2013). That is why the 2D:4D-Ratio can be used as an indirect measure of the ratio of prenatal testosterone levels to estradiol levels.

Sex steroids are important for the establishment of morphological sex differences, and consequently stereotypically female or male behavior (Arnold and Breedlove, 1985). In addition, the brain appears to be influenced by sex steroids in terms of lateralization. According to Geschwind and Galaburda’s (1987) theory, more fetal testosterone leads to compromised development of the left cerebral hemisphere, and increased development of the right hemisphere. Thus, one could conclude that the brain seems to be masculinized by higher prenatal testosterone levels and feminized by lower prenatal testosterone levels (Collaer and Hines, 1995).

In sum, the development of the brain, and therefore behavior and personality, is thought to be influenced by fetal exposure to sex steroids (Geschwind and Galaburda, 1987; Collaer and Hines, 1995). As mentioned above, the 2D:4D-Ratio is related to fetal sex hormones and is therefore often used as an indirect measure of the prenatal testosterone to estradiol ratio (Manning et al., 1998; Lutchmaya et al., 2004). Because of this overlap, an association between the 2D:4D-Ratio and stereotypically female and male personality traits is plausible, and on that basis there is a growing body of research covering this topic (for an overview see Manning, 2011).

Previous findings suggest that a more male/lower 2D:4D-Ratio is associated with more stereotypically male traits, and that a more female/higher 2D:4D-Ratio is associated with more stereotypically female traits (Manning, 2011). For example, Hampson et al. (2008) found significant negative correlations between the 2D:4D-Ratio and sensation seeking, as measured by the SSS by Zuckerman et al. (1978), for both sexes. They were also able to show significant negative correlations between different facets of aggression, measured by the Aggression Questionnaire by Buss and Perry (1992), and the 2D:4D-Ratio of the right hand, but only in females (Hampson et al., 2008). Beyond this, Lindová et al. (2008) examined the relationship between the 2D:4D-Ratio and Cattell’s first-order factors. Among other findings, they showed significant negative correlations between the 2D:4D-Ratio for female participants and Cattell’s emotional stability personality trait (Lindová et al., 2008). Individuals scoring low on emotional stability are typically emotionally reactive and affected by their feelings (Conn and Rieke, 1994). They also found significant negative correlations between the 2D:4D-Ratio and social boldness (Lindová et al., 2008). Individuals scoring high on this factor are socially bold, venturesome, thick skinned, and uninhibited (Conn and Rieke, 1994). In sum, according to the previous research, a smaller 2D:4D-Ratio seems to be associated with levels of relevant traits which are stereotypically male, such as high sensation seeking, high aggression and high emotional stability, and a larger 2D:4D-Ratio seems to be related to stereotypically female levels of relevant traits, such as low aggression and emotional instability (Hampson et al., 2008; Lindová et al., 2008).

One of the most important personality models is the Five-Factor-Model of Personality (sometimes coined the Big Five Model), first discovered by Fiske (1949). After Fiske’s initial work, much research has been undertaken to examine the structure of personality, and the Five-Factor-Model has been confirmed by many studies (Tupes and Christal, 1961/1992; Conley, 1985; Digman and Inouye, 1986; Costa and McCrae, 1988). An often used instrument for measuring the Five-Factor-Model of Personality is the NEO Five Factor Inventory (NEO-FFI), or its longer version the NEO-PI-R, developed by Costa and McCrae (1992). The authors split personality into the five factors of Neuroticism, Extraversion, Openness, Agreeableness and Conscientiousness. Persons scoring high on Neuroticism are typically anxious, nervous, uncertain, easily scared, and stressed (Costa and McCrae, 1992; Borkenau and Ostendorf, 2008). Extraverted people tend to be sociable, outgoing and like stimulation and excitement (Costa and McCrae, 1992; Borkenau and Ostendorf, 2008). The trait of Openness to Experience describes individuals who are eager to experience new things, are imaginative, creative and interested in cultural and intellectual pursuits (Costa and McCrae, 1992; Borkenau and Ostendorf, 2008). Individuals with high scores on Agreeableness are typically altruistic, benevolent, and desire harmony with others (Costa and McCrae, 1992; Borkenau and Ostendorf, 2008). Lastly, trait Conscientiousness relates to individuals being disciplined, neat and typically ambitious (Costa and McCrae, 1992; Borkenau and Ostendorf, 2008). Sex differences in these five facets have been observed in many studies. Findings have been heterogeneous, although differences on Neuroticism are often replicated; it has been frequently reported that females tend to score higher on Anxiety as a sub-facet of Neuroticism, and on Neuroticism in total (Feingold, 1994; Weisberg et al., 2011). In addition, Weisberg et al. (2011) have reported higher scores on Agreeableness for females. For Extraversion, the pattern was more complicated, with males reporting higher scores on the Assertiveness facet of Extraversion, but females reporting higher total Extraversion scores (Feingold, 1994; Weisberg et al., 2011). Finally, sex differences on the Big Five factors of Openness and Conscientiousness have been mixed across studies, and are therefore unclear (Weisberg et al., 2011).

In sum, scores on Big Five personality factors and the 2D:4D-Ratio, differ between and within sexes (Manning et al., 1998; Weisberg et al., 2011). Given previous findings already suggest a correlation between the 2D:4D-Ratio and stereotypic male and female personality factors (for example Hampson et al., 2008; Lindová et al., 2008), an association between 2D:4D-Ratio and the Big Five personality traits might be expected. Indeed, Fink et al. (2004) examined the association between the 2D:4D-Ratio and the Big Five personality factors, measured using the NEO-FFI, in a sample consisting of 50 males and 70 females. Positive correlations between the 2D:4D-Ratio and Neuroticism were found for the right hand in females and in the whole sample, but not in males alone. Also, a significant negative correlation was found between the 2D:4D-Ratio and Agreeableness in females for the right hand. Correlations between the 2D:4D-Ratio and Extraversion were mixed, and associations between the 2D:4D-Ratio and Openness and Conscientiousness were negative, but not significant for both sexes (Fink et al., 2004). Another study by Lippa (2006) arrived at different results, but used instead the classic NEO-FFI, another measure assessing the Five-Factor-Model of Personality (a 44 item inventory by John and Srivastava, 1999). Lippa (2006) observed a weak positive association between digit ratios and Extraversion, and a negative association between digit ratios and Openness to Experience. Given the heterogeneity of findings in the field with respect to the link between 2D:4D-Ratio and the Five-Factor-Model of Personality, we aimed to extend this existing research by collecting 2D:4D-Ratios in a German and a Chinese sample, alongside the NEO-FFI, used to assess individual differences in personality. Given the findings summarized above, we expected a significant positive correlation between the 2D:4D-Ratio and Neuroticism, with this effect specific to the female participants in both samples.

## Materials and Methods

### Participants

Participants were recruited at the University of Civil Engineering and Architecture in Beijing, China and at Ulm University in Ulm, Germany. Most of the participants were students. Before filling in the online questionnaires, participants gave electronic approval to participate in the study. Afterward, all participants were invited to give their written consent and scan their hands for measuring the 2D:4D-Ratio. The study was approved by the local ethics committee at Ulm University, Ulm, Germany.

After exclusion of all participants who ever had broken fingers, 448 (*n*_male_ = 125, *n*_female_ = 323) participants provided complete data for the study. Of these, 35 males (*M* = 19.14, *SD* = 0.77) and 43 females (*M* = 20.60, *SD* = 5.34) were recruited in China. Unfortunately, the variable about ethnicity was only recorded in the German sample – see also limitation section. Nevertheless, with some certainty, we can characterize the Chinese sample as mainly Han Chinese (by memory recall). In Germany 90 white male participants (*M* = 23.19, *SD* = 3.50) and 280 white female participants (*M* = 22.51, *SD* = 2.67) were recruited. There was a significant difference in age between the two national samples [*F*(1,444) = 55.11, *p* < 0.001], with the Chinese participants being younger (*M* = 19.95, *SD* = 4.05 vs. *M* = 22.68, *SD* = 2.90). The effect of sex on age did not reach significance [*F*(1,444) = 0.96, *p* = 0.327], but the interaction term of Sex^∗^Nation on age was found to be significant [*F*(1,444) = 7.09, *p* = 0.008]. In the Chinese sample females were older than males, but in the German sample males were older than females. Mean age for the whole sample was 22.20 years (*SD* = 3.29). The distribution of sex differed significantly between nation [χ^2^(1, *N* = 448) = 13.52, *p* < 0.001], with a greater number of females compared to males in the German subsample (and nearly the same number of male and female participants in the Chinese subsample).

### Measuring 2D:4D-Ratio

Scans of both hands of every participant were taken using CANON-Scanners. The length of index (2^nd^) finger and ring (4^th^) finger was measured digitally by two independent raters using the software GIMP 2.8^1^. 2D:4D-Ratios for each hand were calculated by each rater by dividing the length of the index (2^nd^) finger by the length of the ring (4^th^) finger of the same hand. Thus, four ratios were calculated for each person: two for the right hand, and two for the left hand. Interrater correlations for 2D:4D-Ratios were *r* = 0.92 (*p* < 0.001) for the left and *r* = 0.88 (*p* < 0.001) for right hand measures for the Chinese sample. For the German sample, correlations were *r* = 0.88 (*p* < 0.001) for left and *r* = 0.87 (*p* < 0.001) for right hand 2D:4D measures. Because of the high interrater-reliabilities, the mean ratios of both raters were calculated for each hand for each participant. So, one 2D:4D-Ratio for the left hand and one 2D:4D-Ratio for the right hand per participant remained for further analyses.

### Personality

To measure personality factors and replicate the study of Fink et al. (2004) as precisely as possible, the NEO-FFI Revised (Costa and McCrae, 1992) was used. It is a 60-item questionnaire measuring the five personality factors of Neuroticism, Extraversion, Openness, Agreeableness and Conscientiousness, with each scale consisting of 12 items. In the Chinese subsample, the Chinese version of the NEO-FFI was used. It has been previously shown to be reliable by Melchers et al. (2016). For the German subsample, the German version of the NEO-FFI (Costa and McCrae, 1992), translated by Ostendorf and Angleitner (2003), was used. Both samples received the questionnaires online.

In the Chinese sample, reliabilities of the five scales ranged between α = 0.55 for Openness and α = 0.84 for the Neuroticism scale. Reliabilities of the other scales were α = 0.59 for Agreeableness, α = 0.64 for Conscientiousness and α = 0.70 for Extraversion. In this study, the reliabilities for the German version of the NEO-FFI ranged between α = 0.76 for Openness and α = 0.85 for Neuroticism and Conscientiousness (also α = 0.85). The reliability of Extraversion and Agreeableness were both 0.79. Thus, all reliabilities were acceptable, although it should be noted that the reliabilities for the Chinese version of the NEO-FFI Openness and Agreeableness scales were relatively low, and results presented using these scales should be treated with some caution.

### Statistical Analysis

All statistical analyses were done using the software SPSS 21 (IBM). First, all variables of interest, separately for each nation, were tested for normality because of differences in the reliabilities of the tests in distinct languages. Statistical normality tests will show a significant departure from normality with large sample sizes with even small deviations from normality, so normality was checked visually (Bortz, 2005; Ghasemi and Zahediasl, 2012). After a visual inspection of the histograms of the scores of the NEO-FFI and the mean 2D:4D-Ratios of both hands split by nation, the normal distribution for all of these variables was assumed. Furthermore, normal distributions could be assumed according to the central limit theorem, because sample sizes of all subsamples were larger than 30 (Bortz, 2005). In addition, the skewness and kurtosis of nearly all scores of NEO-FFI variables and 2D:4D-Ratios were lower than 1 in all subsamples, which also indicates normal distribution (split by nation and sex) (Miles and Shevlin, 2001)^2^.

A multivariate ANOVA was used to identify effects of sex and nation, as well as the interaction of sex and nation (independent variables), on 2D:4D-Ratios and Big Five factors (dependent variables). After this, independent-sample *t*-tests were conducted to examine differences in these variables across males and females in the different nations. A *t*-test was chosen to replicate the analytic approach of Fink et al. (2004) as precisely as possible. Lastly, Pearson’s Product-Moment Correlations were calculated for the whole sample, as well as for each sample and sex separately, to explore associations between 2D:4D-Ratios and sum scores of each Big Five personality factor. As in the study of Fink et al. (2004), correlations were conducted using a two-tailed test for all personality factors. Even if we hypothesized above that the 2D:4D-Ratio and Neuroticism would be positively correlated, only two-tailed tests are reported because of some negative correlations between these two variables – see also result section. Furthermore confidence intervals for the correlations between the 2D:4D-Ratios and the Big Five personality factors are presented. These were calculated with the help of bootstrap analysis (Haukoos and Lewis, 2005). Bootstrap analysis provide the reader with robust estimators, even in cases of small sample sizes where the assumption of normal distribution can only be approximated.

## Results

### Differences in 2D:4D-Ratios and Personality Factors between Sexes and Nations

In line with the analytic approach used by Fink et al. (2004), we examined differences in 2D:4D-Ratios and the Big Five personality factors across the variables sex and nation (they also included samples from different countries and both sexes). Using a multivariate ANOVA, significant effects (*p* < 0.01) of sex were found on Agreeableness [*F*(1,444) = 10.73, *p* = 0.001] and 2D:4D-Ratio [left: *F*(1,444) = 7.06, *p* = 0.008; right: *F*(1,444) = 9.91, *p* = 0.002], with females tending to have higher scores than males on all three variables. Significant effects (*p* < 0.01) of nation were found on Openness [*F*(1,444) = 9.94, *p* = 0.002], Agreeableness [*F*(1,444) = 13.78, *p* < 0.001] and Conscientiousness [*F*(1,444) = 39.33, *p* < 0.001], with the German sample having higher scores than the Chinese sample. There were no significant differences between nations in the 2D:4D-Ratios [left: *F*(1,444) = 0.95, *p* = 0.331; right: *F*(1,444) = 1.59, *p* = 0.207]. The effect of the Sex^∗^Nation interaction was significant (*p* < 0.01) on Neuroticism [*F*(1,444) = 7.00, *p* = 0.008; Chinese sample: males > females (descriptively); German sample: males < females], Agreeableness [*F*(1,444) = 6.64, *p* = 0.010; Chinese sample: males < females (descriptively); German sample: males < females] and Conscientiousness [*F*(1,444) = 6.88, *p* = 0.009; Chinese sample: males > females (descriptively); German sample: males < females]. There were no effects of the Sex^∗^Nation interaction on the 2D:4D-Ratios [left: *F*(1,444) = 0.26, *p* = 0.611; right: *F*(1,444) = 0.69, *p* = 0.408]. Because of differences in personality factors between nations (and the sex by nation interaction effects), *t*-tests comparing males and females regarding the Big Five factors were calculated for the whole sample, as well as split by nation.

As seen in **Table 1**, significant differences between sex for 2D:4D-Ratios for both hands were found, with males having smaller ratios than females. Men also showed significantly smaller values for Neuroticism, Openness, Agreeableness, and Conscientiousness when analyzing the full sample.

**Table 1:** Differences in digit ratios and Big Five personality factors between sexes in the full sample.

	Mean (*SD*)		
	Males (*n* = 125)	Females (*n* = 323)	*t*	*p*
2D:4D left	0.97 (0.03)	0.98 (0.03)	-3.80	<0.001^∗∗∗^
2D:4D right	0.97 (0.03)	0.99 (0.03)	-4.67	<0.001^∗∗∗^
Neuroticism	2.63 (0.61)	2.79 (0.64)	-2.38	0.018^∗^
Extraversion	3.38 (0.50)	3.44 (0.51)	-1.01	0.313
Openness	3.42 (0.48)	3.54 (0.56)	-2.13	0.034^∗^
Agreeableness	3.49 (0.48)	3.81 (0.49)	-6.37	<0.001^∗∗∗^
Conscientiousness	3.57 (0.56)	3.83 (0.54)	-4.54	<0.001^∗∗∗^

*Values are calculated for the full male and full female sample across both nations. ^∗^*p* < 0.05, ^∗∗^*p* < 0.01, ^∗∗∗^*p* < 0.001, two-tailed*.

As seen in **Table 2**, no significant sex differences in the Big Five factor were found in the Chinese sample. It should be noted that this sample was much smaller compared to the German sample, and so significant differences will be harder to detect. In the German sample, there were significant differences in the 2D:4D-Ratios of both hands as well as in the personality factors Neuroticism, Openness, Agreeableness, and Conscientiousness across sex, with higher scores for females than males on all of these factors.

**Table 2:** Differences in Big Five personality factors between sexes split by Nation.

	China				Germany			
	Mean *(SD)*				Mean *(SD)*		
	Males (*n* = 35)	Females (*n* = 43)	*T*	*p*	Males (*n* = 90)	Females (*n* = 280)	*t*	*P*
Neuroticism	2.87 (0.62)	2.71 (0.55)	1.23	0.223	2.54 (0.58)	2.80 (0.65)	-3.44	0.001^∗∗^
Extraversion	3.35 (0.43)	3.47 (0.43)	-1.19	0.239	3.40 (0.52)	3.43 (0.53)	-0.59	0.559
Openness	3.38 (0.37)	3.22 (0.42)	1.70	0.094	3.44 (0.52)	3.59 (0.56)	-2.28	0.023^∗^
Agreeableness	3.44 (0.41)	3.48 (0.39)	-0.47	0.637	3.51 (0.51)	3.87 (0.49)	-6.04	<0.001^∗∗∗^
Conscientiousness	3.39 (0.34)	3.32 (0.40)	0.91	0.368	3.64 (0.62)	3.91 (0.51)	-4.15	<0.001^∗∗∗^

*^∗^*p* < 0.05, ^∗∗^*p* < 0.01, ^∗∗∗^*p* < 0.001, two-tailed*.

### The 2D:4D-Ratio and Personality

As the 2D:4D-Ratio of the left and right hand differed significantly between sexes, correlations and confidence intervals are reported for the whole sample, as well as for males and females separately. In contrast to Fink et al. (2004), data were also analyzed separately for each nation because of significant differences in many of the NEO-FFI-scores. Results for the whole sample are shown in **Table 3**. Results for each nation are shown in **Tables 4** and **5**.

**Table 3:** Pearson’s Product-Moment Correlations of digit ratios and NEO-FFI scores and the corresponding confidence intervals for the whole sample.

	Whole sample (left)	Whole sample (right)	Males (left)	Males (right)	Females (left)	Females (right)
Neuroticism	0.11^∗^	0.09	-0.10	-0.15	0.17^∗∗^	0.15^∗∗^
95%-CI	[0.01; 0.21]	[0.00; 0.19]	[-0.29; 0.09]	[-0.33; 0.04]	[0.07; 0.25]	[0.04; 0.24]
Extraversion	-0.04	-0.02	-0.04	0.00	-0.05	-0.04
95%-CI	[-0.13; 0.05]	[-0.11; 0.08]	[-0.20; 0.13]	[-0.17; 0.16]	[-0.16; 0.06]	[-0.14; 0.08]
Openness	0.08	0.05	0.15	0.12	0.03	-0.01
95%-CI	[-0.01; 0.17]	[-0.05; 0.13]	[-0.06; 0.33]	[-0.05; 0.28]	[-0.13; 0.05]	[-0.10; 0.09]
Agreeableness	0.02	0.06	-0.05	-0.03	-0.03	0.01
95%-CI	[-0.07; 0.10]	[-0.02; 0.15]	[-0.22; 0.12]	[-0.20; 0.15]	[-0.08; 0.13]	[-0.08; 0.11]
Conscientiousness	0.02	0.06	0.13	0.06	-0.08	-0.01
95%-CI	[-0.07; 0.12]	[-0.03; 0.16]	[-0.03; 0.29]	[-0.12; 0.23]	[-0.13; 0.08]	[-0.11; 0.10]

*^∗^*p* < 0.05, ^∗∗^*p* < 0.01, ^∗∗∗^*p* < 0.001, two tailed. *N* = 448, *n*_*male*_ = 125, *n*_*female*_ = 323. Confidence intervals (CIs) were calculated using botstrap analysis (1000 samples, Bias corrected and accelerated)*.

**Table 4:** Pearson’s Product-Moment Correlations of digit ratios and NEO-FFI scores and the corresponding confidence intervals for Chinese sample.

	Whole sample (left)	Whole sample (right)	Males (left)	Males (right)	Females (left)	Females (right)
Neuroticism	0.07	-0.02	-0.30	-0.31	0.42^∗∗^	0.26
95%-CI	[-0.18; 0.31]	[-0.27; 0.23]	[-0.57; -0.05]	[-0.65; -0.01]	[0.11; 0.66]	[-0.06; 0.56]
Extraversion	-0.05	0.08	0.09	0.19	-0.19	-0.04
95%-CI	[-0.27; 0.16]	[-0.14; 0.26]	[-0.26; 0.48]	[-0.15; 0.50]	[-0.46; 0.07]	[-0.33; 0.25]
Openness	0.15	0.07	0.11	0.01	0.23	0.15
95%-CI	[-0.06; 0.35]	[-0.11; 0.26]	[-0.23; 0.42]	[-0.27; 0.28]	[-0.13; 0.52]	[-0.15; 0.44]
Agreeableness	-0.18	-0.02	-0.14	0.08	-0.22	-0.11
95%-CI	[-0.38; 0.03]	[-0.22; 0.20]	[-0.44; 0.16]	[-0.26; 0.41]	[-0.48; 0.09]	[-0.36; 0.19]
Conscientiousness	-0.08	0.01	0.18	0.25	-0.21	-0.12
95%-CI	[-0.30; 0.15]	[-0.20; 0.22]	[-0.21; 0.51]	[-0.10; 0.54]	[-0.53; 0.12]	[-0.40; 0.18]

*^∗^*p* < 0.05, ^∗∗^*p* < 0.01, ^∗∗∗^*p* < 0.001, two tailed. *n* = 78, *n*_*male*_ = 35, *n*_*female*_ = 43. Confidence intervals were calculated using bootstrap analysis (1000 samples, Bias corrected and accelerated)*.

**Table 5:** Pearson’s Product-Moment Correlations of digit ratios and NEO-FFI scores and the corresponding confidence intervals for German sample.

	Whole sample (left)	Whole sample (right)	Males (left)	Males (right)	Females (left)	Females (right)
Neuroticism	0.12^∗^	0.12^∗^	-0.02	-0.08	0.13^∗^	0.13^∗^
95%-CI	[0.03; 0.22]	[0.02; 0.22]	[-0.26; 0.24]	[-0.30; 0.17]	[0.02; 0.23]	[0.02; 0.23]
Extraversion	-0.04	-0.03	-0.07	-0.06	-0.03	-0.04
95%-CI	[-0.13; 0.05]	[-0.13; 0.05]	[-0.27; 0.14]	[-0.26; 0.14]	[-0.15; 0.08]	[-0.15; 0.08]
Openness	0.05	0.02	0.16	0.14	-0.01	-0.05
95%-CI	[-0.06; 0.16]	[-0.09; 0.12]	[-0.07; 0.37]	[-0.08; 0.34]	[-0.13; 0.12]	[-0.17; 0.08]
Agreeableness	0.03	0.05	-0.03	-0.07	-0.03	0.00
95%-CI	[-0.08; 0.14]	[-0.04; 0.14]	[-0.23; 0.19]	[-0.27; 0.14]	[-0.13; 0.08]	[-0.10; 0.11]
Conscientiousness	0.00	0.03	0.11	0.02	-0.10	-0.04
95%-CI	[-0.11; 0.12]	[-0.08; 0.13]	[-0.06; 0.27]	[-0.18; 0.22]	[-0.23; 0.03]	[-0.15; 0.07]

*^∗^*p* < 0.05, ^∗∗^*p* < 0.01, ^∗∗∗^*p* < 0.001, two tailed. *n* = 370, *n*_*male*_ = 90, *n*_*female*_ = 280. Confidence intervals (CIs) were calculated using bootstrap analysis (1000 samples, Bias corrected and accelerated)*.

As seen in **Table 3**, for the whole sample significant positive correlations were only found between 2D:4D-Ratio of the left hand and Neuroticism. There were no significant correlations found in the whole male sample, but significant positive correlations were found between 2D:4D-Ratio of both hands and Neuroticism in the whole female sample. All Pearson’s Correlations could be observed in the corresponding 95% confidence interval. In addition, the lower as well as the upper limit of confidence intervals belonging to significant positive correlations were positive. In detail, these confidence intervals did not include the 0 (hence CI > 0) and thus supported the significance of the corresponding positive correlations. All confidence intervals corresponding to non-significant correlations included negative as well as positive values. In line with our argumentation, this supported the non-significance of these correlations. Within the Chinese sample the only significant correlation was found for the female subsample between 2D:4D-Ratio of the left hand and Neuroticism (s. **Table 4**).

As seen in **Table 5**, there were positive correlations found between 2D:4D-Ratios of both hands and Neuroticism for the whole sample. But these associations were largely driven by the positive correlations in the female subsample. Again all significances and non-significances of the correlations were supported by the range of the corresponding confidence intervals as described above.

For the whole male sample, as well as the male subsamples from the different nations, small to moderate negative correlations between 2D:4D-Ratio and Big Five personality factor Neuroticism were found. As an additional note: even if the Pearson’s Correlations were not significant in these cases a closer look on the confidence intervals reveals some interesting further insight into the data set. The confidence intervals of these correlations were strongly negative in the Chinese male subsample. Despite this, these latter results should clearly be treated with caution, as we hypothesized a positive relationship between the 2D:4D-Ratio and Neuroticism, not an inverse relationship.

In sum, we can conclude that the clearest finding from this data is that is that the 2D:4D-Ratio of the left hand is significantly and positively associated with Neuroticism in females, but not in males. This could be observed for both the group of females in China and Germany.

## Discussion and Conclusion

In support of the findings from Fink et al. (2004) and findings from many other previous studies, significant differences in 2D:4D-Ratios of both hands were found between sexes, with males having smaller ratios than females (Manning, 2011). These results support the role of prenatal sex steroids on the development of fingers. In contrast to Fink et al. (2004), in this study females had significantly higher scores on Conscientiousness, Neuroticism and Openness, as well as Agreeableness, when considering the whole sample. These results for Neuroticism and Agreeableness are in line with previous findings (Feingold, 1994; Weisberg et al., 2011). The differences between sexes found in the whole sample were, however, largely driven by significant differences between males and females in the German subsample. In the Chinese sample no significant sex differences on any of the Big Five factors were found, although as noted above the results for the Chinese subsample should be viewed cautiously, as internal consistencies for the NEO-FFI factors were relatively low and the sample size was much smaller than the German subsample^3^. We should note though, that the Cronbach’s alpha for Neuroticism in the Chinese subsample was good (α = 0.84), so therefore we can have more confidence in the main analyses relating to the correlation between 2D:4D-Ratio and Neuroticism. Despite these methodological issues, supportive evidence for the different results on sex and personality comes from a study published Schmitt et al. (2008), where sex differences in Big Five factors were examined in 55 different countries. There the researchers found greater sex differences in Western and Eastern Europe than in South and East Asian regions. The variation in gender differences in personality were explained there by evolutionary theories and gene^∗^environment-interactions (Schmitt et al., 2008).

Partly in line with Fink et al. (2004), we observed positive correlations between 2D:4D-Ratios of both hands and Neuroticism in females from the full sample, and from the German subsample. Within the Chinese female subsample, significant correlations of 2D:4D-Ratio and Neuroticism were found only for the left hand. The here discovered stronger correlations between the left 2D:4D-Ratio and Neuroticism than between the right 2D:4D-Ratio and Neuroticism is in line with findings of Austin et al. (2002). In this study correlations between 2D:4D-Ratio and personality traits like Neuroticism where measured using the Eysenck Personality Questionnaire – Revised (Eysenck et al., 1985; Austin et al., 2002). But in contrast to our findings and the findings of Austin et al. (2002), Fink et al. (2004) only found a significant positive correlation between the 2D:4D-Ratio of the right hand and Neuroticism in the whole sample and the female-only sample. In sum, the findings on differences in the correlations of the 2D:4D-Ratio of the left/right hand and Neuroticism are inconsistent (Austin et al., 2002; Fink et al., 2004; Lindová et al., 2008). At this stage of research it is not clear if overall one (the left or the right) 2D:4D-Ratio is stronger associated with personality traits like Neuroticism than the other one. Nonetheless, even if results suggest associations between 2D:4D-Ratio and Neuroticism for different hands, in females a general link – more specifically a positive correlation – between the 2D:4D-Ratio and Neuroticism appear to be robust (for example: Fink et al., 2004; Lindová et al., 2008).

In contrast to Fink et al. (2004), who found significant negative associations between right hand 2D:4D-Ratio and Agreeableness in females, in this study no significant negative correlations were found. The study by Lippa (2006) showed different results compared to our study, but we should note that they used a different measure of the Five-Factor-Model. Future studies should therefore aim to include both measures of personality (NEO-FFI and John and Srivastavas’s Big Five Questionnaire) in one study to help clarify the findings across the two inventories. As a basis for future research endeavors, we have also provided results of the link between the 2D:4D ratio and Jaak Panksepp’s primary emotional systems (Davis et al., 2003) for the German subsample. It has been proposed that individual differences in these primary emotional systems are anchored in the ancient mammalian brain and so could represent the foundation of the Big Five (Davis and Panksepp, 2011). Please see the results provided in the Supplementary Material.

We were not able to show any significant correlations for either of the male samples. This is in line with previous findings reporting smaller effect sizes in the context of 2D:4D research in males compared to females (for example Fink et al., 2004; Lippa, 2006; Hampson et al., 2008; Manning, 2011). The reason for these findings is unclear at this stage of the research and different researchers have suggested different explanations for these small effect sizes (Hampson et al., 2008; Manning, 2011). Despite not being significant, though, negative correlations between 2D:4D-Ratios and Neuroticism for the two male subsamples, and for the whole male sample, were found. In the Chinese male subsample the confidence intervals carved out by bootstrap analysis strongly encourage these negative correlations. That the correlations are not significant in this subsample could support the idea that the non-significant result in the Chinese sample is due to power issues. But it is also possible that the approximation of the normal distribution is not accurate for this subsample. Nevertheless these results indicate more fetal testosterone is associated with higher Neuroticism in males (especially in the Chinese male subsample), but with lower Neuroticism in females. These opposing associations are of potentially great interest, and there is a need for further research in this regard. Methodological limitations with the current data limit our ability to further examine this issue here, but future studies should endeavor to examine this issue more thoroughly.

Several limitations with the current study should be mentioned. Firstly, the numbers of participants in each of the subsamples from the different countries, as well as the distribution of sexes within each sample, were unequal. There were more German participants than Chinese participants in the study. More specifically, the German subsample had a greater proportion of (three times more) females than males; this is due to the gender distribution within psychology classes in Germany. Therefore, significant results in the female, but not male subsamples, could be explained on this basis. Secondly, the reliabilities of the Chinese version of the NEO-FFI, especially for the Openness and Agreeableness scales, were rather low. This could be due to the fact that the Chinese language is a symbolic language and therefore word for word translations are difficult. In contrast to our data, Melchers et al. (2016) found acceptable reliabilities for the five factors, with reliabilities lying between 0.61 and 0.86. It is possible that the small Chinese sample size is the reason for the low reliabilities found in some of the present personality dimensions in the present study (the main finding with Neuroticism is not affected by this issue). If the sample sizes had been larger, we potentially would have been able to find higher reliabilities and also would have been able to find differences in the Big Five factors between sexes in the Chinese, as well as the German, subsample. Another point which has to be discussed about this study is that 2D:4D-Ratios were measured indirectly. This means scans of both hands of every participant were taken using CANON-Scanners. Afterward the length of index (2^nd^) finger and ring (4^th^) finger was measured digitally from these scans. Compared to this procedure direct measurement implies that the length of the fingers is directly measured from the hands of the participants. In several studies the means of indirectly measured 2D:4D-Ratios were found to be smaller than means of directly measured 2D:4D-Ratios (Manning et al., 2005; Kim and Cho, 2013; Xu and Zheng, 2015). But in contrast to these findings other studies suggest indirectly measured 2D:4D-Ratios to be bigger than directly measured 2D:4D-Ratios (Voracek and Dressler, 2006; Dressler and Voracek, 2011). Also the sex differences between 2D:4D-Ratio were found to be stronger when calculated from indirect measurements (Manning et al., 2005; Dressler and Voracek, 2011; Kim and Cho, 2013). So findings of this study according to sex differences in 2D:4D-Ratios should be treated cautiously. Nevertheless in our opinion measuring length of the digits indirectly was the best method to replicate the findings of Fink et al. (2004), which were also based on indirect measurements^4^.

Finally, and in line with previous research, the correlations between Neuroticism and the digit ratio are significant, but not particularly high. Obviously, many other factors are involved in shaping the neurotic personality trait.

We should note that further research will be necessary to support and extend the present results. One interesting starting point for future research endeavors would be the investigation of interactions between exposure to fetal sex steroids and environmental factors such as parental upbringing and socio-economic status, and their combined effects on personality. We believe that this hitherto unexplored approach could shed light on the heterogeneous findings in the 2D:4D-Ratio and personality literature (Putz et al., 2004). With regard to Neuroticism and its link with mental disorders, more research is necessary to examine how, and under which circumstances, the neurotic trait has an impact on mental health. This research direction is justified by the many previous results suggesting that Neuroticism is robustly associated with (and a predisposing factor for) many mental disorders and physical health problems, as well as subjective well-being (for overviews see for example DeNeve and Cooper, 1998; Lahey, 2009). In conclusion, more “female” hands appear to be robustly associated with higher Neuroticism in females, underlining the influence of both prenatal testosterone and estrogen on human personality.

## Author Contributions

CM and ML designed the study. CS wrote the manuscript and did the statistical analysis. CS, CM, RS, and BL collected the German data. ML collected the Chinese data. LW, ML, and CS analyzed the 2D:4D marker from the hands. ÉD, AC, RS, BL critically revised the draft of the manuscript.

## Conflict of Interest Statement

The authors declare that the research was conducted in the absence of any commercial or financial relationships that could be construed as a potential conflict of interest.

## Footnotes

1 https://www.gimp.org/.

2 In the Chinese male sample the kurtosis of the distribution of the right 2D:4D-Ratio was greater than 1. But because the kurtosis was still lower than two times the corresponding standard error, normal distribution was assumed (Miles and Shevlin, 2001). In the German female subsample the kurtosis of the distribution of the Agreeableness scale was greater than 1 and greater than two times the corresponding standard error. But because the sample size of this subsample was *n* = 280, according to the central limit theorem normal distribution was also assumed for this subsample (Bortz, 2005).

3 The sample size of the initial study by Fink et al. (2004) was also rather small.

4 A last issue not mentioned is handedness: We assessed handedness in the German, but not the Chinese sample. In Germany only a small number of the sample indicated to be left-handed in terms of writing with the left hand (*n* = 28 consisting of 12 males and 16 females). Excluding those participants from the analysis in the German sample did not change the main results. Also the correlations between 2D:4D-Ratios of both hands and personality traits didn’t differ between people of different handedness (indicated by writing). In general, due to the smaller prevalence of left handedness in the population (which is best assessed with the Edinburgh Handedness Inventory), it is hard to answer the question if studies on 2D:4D and personality are influenced by this factor.
