# Supplementary material for: The 2D:4D-Ratio and Neuroticism Revisited: Empirical Evidence from Germany and China
Source: Front Psychol. 2016 Jun 10;7:811. doi: 10.3389/fpsyg.2016.00811 (PMC4901036; doi:10.3389/fpsyg.2016.00811)
Supplement: Supplementary file 1 [file Presentation_1.PDF]

Supplementary Material

The 2D:4D-Ratio and Neuroticism revisited:

Empirical evidence from Germany and China

Cornelia Sindermann[1*], Mei Li[2], Rayna Sariyska[1], Bernd Lachmann[1], Éilish Duke[3], Andrew Cooper[3] Lidia Warneck[1] & Christian Montag[1*]

*to whom correspondence should be addressed

Cornelia Sindermann & Prof. Dr. Christian Montag
Department of Molecular Psychology
Insitute of Psychology and Education
Ulm University
Helmholtzstr. 8/1
89081 Ulm

cornelia.sindermann@uni-ulm.de
christian.montag@uni-ulm.de

Supplement

The Affective Neuroscience Personality Scale (ANPS) is a questionnaire measuring individual differences in six primary emotions based on six distinct groups of brain affective systems emerging from affective neuroscience research (Davis et al., 2003; Panksepp, 1998). The six neurally based networks are labeled PLAY, SEEK, CARE (positive circuitry), FEAR, ANGER and SADNESS (negative circuitry). These terms are written in capital letters so as not to confound them with terms in the psychological literature that use the same name. Each of these six scales consists of 14 Items. One additional scale called Spirituality has been included to explore therapeutical issues (Davis et al., 2003). The questionnaire consists of 110 items answered on a four Likert scale ranging from strongly agree to strongly disagree (Davis et al., 2003). We recommend to use the scaling from strongly disagree to strongly agree in the future though (as in the ANPS revision, Davis & Panksepp, 2011), because it is more intuitive to answer from the participants perspective and easier with respect to coding.

The scales are defined as follows: The playfulness scale is conceptualized as an individual having fun versus being serious. Individuals scoring high on PLAY enjoy humor, laughter and describe themselves as happy and joyful. Individuals scoring high on the SEEK scale are reported to be curious, like to explore the environment and their surroundings, and positively anticipate new experiences. High scores on the CARE scale are linked with being softhearted towards animals and people in need. Caring persons feel empathy and like to take care of others. High scorers on FEAR are defined as being anxious, worrying a lot and ruminating about past decisions. It also describes people who are typically not courageous.

People scoring high on the ANGER scale can be described as being hotheaded, easily irritated and frustrated. They tend to express their anger verbally and physically. The SADNESS scale is about feeling lonely, crying frequently and thinking about past relationships (Davis et al., 2003).

Another 12 other Items comprise the "Spirituality" scale which measures the degree of feeling "connected" to humanity and creation as a whole (Davis et al., 2011). 14 further items are "filler questions" (Davis et al., 2003). In the present study, reliabilities of the scales were α = .71 (SEEK), α = .74 (SADNESS), α = .79 (PLAY), α = .80 (CARE & ANGER), α = .84 (Spirituality) and α = .87 (FEAR). Correlations of the ANPS scales and 2D:4D-Ratios of both hands for the whole German sample, and separately by male and female within the German sample, are shown in table 6. This questionnaire was not administered in the Chinese sample.

Supplementary Table 1: Correlations of 2D:4D-Ratios and ANPS scales for the German sample.

	Whole sample (left)	Whole sample (right)	Males (left)	Males (right)	Females (left)	Females (right)
PLAY	-.04	-.07	-.02	.04	-.05	-.11
SEEK	.07	.05	.07	.08	.05	.02
CARE	.01	.05	-.11	-.12	-.06	-.02
FEAR	.10	.12*	(-).00	-.05	.09	.12*
ANGER	.07	.09	.06	.07	.05	.07
SADNESS	.09	.14**	.03	-.05	.05	.12*
Spirituality	-.02	.02	-.06	.01	-.02	.00

** $p < .01$, two-tailed, *$p < .05$,two-tailed. $n = 370$, $n_{male} = 90$, $n_{female} = 280$.

The results in Table 6 show that more female hands are significantly and positively associated with FEAR and SADNESS scores. The effect is clearly driven by the female subsample. As SADNESS and FEAR could represent the phylogenetically old bases of neuroticism (see the large positive correlations with Neuroticism in the Davis et al., 2003 study), the findings in this supplement support the main observations of the present study, namely that higher negative emotionality is linked to less prenatal testosterone in females. Of note, correlations between FEAR, SADNESS and Neuroticism in the present study are as follows:

Supplementary Table 2: Correlations between FEAR, SADNESS and Neuroticism.

	Correlations without controlling for gender			Correlations controlling for gender		
	FEAR	SADNESS	Neuroticism	FEAR	SADNESS	Neuroticism
FEAR						
SADNESS	.68**			.66		
Neuroticism	.78**	.70**		.77	.69	

** $p < .01$ two-tailed. $n = 370$.
